# The effect of skin traction on pain relief in patients with isolated intertrochanteric fractures, a randomized clinical trial

**DOI:** 10.1186/s12891-023-06135-0

**Published:** 2023-01-12

**Authors:** Sara Kheiri, Hossein Akbari Aghdam, Mehdi Motififard, Navid Gharib Gashteh Shahi, Mohammad Saleki Mehrjardi, Tayebe Rezaei

**Affiliations:** Isfahan University of Medical Sciences, Isfahan, Iran

**Keywords:** Skin traction, Visual Analogue Scale, VAS, Analgesics, Hip fractures, RCT, Randomized controlled trial

## Abstract

**Background:**

Hip fractures are common in elderly patients. The surgery is usually delayed due to underlying conditions, and pain control is crucial while the patient is cleared for surgery. In this randomized controlled trial (RCT) study, we hypothesized that the application of skin traction in patients with intertrochanteric fracture does not significantly change the Visual Analogue Score (VAS) of pain.

**Methods:**

This is a prospective, single institution, parallel randomized controlled trial. Two hundred and twenty-nine patients with isolated intertrochanteric fractures were enrolled in the study. Patients with neurologic issues, drug addiction, scars or swelling, or vascular issues at the site of skin traction application were excluded from the study. Patients were divided into two groups: group A included 97 patients, and group B included 95 patients. Skin traction was applied for group A, while only a soft pillow was put beneath the patients’ knees in the other group. The VAS score was measured after the diagnosis, two hours before the operation, and 24 h after the surgery. The morphine dosage administered per day was documented for both groups.

**Results:**

After excluding patients with postoperative delirium, 154 patients (55 males and 99 females) with isolated intertrochanteric fractures (69 right-sided and 85 left-sided), and a mean age of 70 ± 10 remained in the study. There were no significant differences between the two groups regarding age, gender, and mean time from injury to admission (*P* > .05). The mean VAS score measures and morphine dosage administered per day were not significantly different between the two groups (*P* > .05). Both groups experienced significant pain relief 24 h postoperatively (*P* < .001).

**Conclusion:**

Pre-operative skin traction application affected neither the patients' VAS scores nor the mean morphine dosage per day in patients with isolated intertrochanteric fractures. Our data does not support the routine application of pre-operative skin traction in patients with intertrochanteric fractures.

**Trial registration:**

The project was registered in the Iranian Registry of Clinical Trials (registration reference: IRCT20180729040636N3, registration date: 01/07/2020).

**Level of evidence:**

1.

## Background

Hip fractures are common in orthopedic trauma, and current practice and literature suggests improved outcomes with early operation [1]. Intertrochanteric fractures account for almost half of total hip fractures [2]. Patients with intertrochanteric fractures are usually older and in poorer health compared to patients with other types of fractures [3]. Due to these patients’ pre-existing background diseases, the operation is usually done with delay. Therefore, pain control between the admission time and surgery in these patients is very important [4].

In addition, patients with severe pain during hospitalization are at higher risk for experiencing delirium and other post-operative complications [5, 6]. Opioid administration is introduced as the principal preoperative (and postoperative) pain management option for this group of patients; Despite the fact that opioids are advised to be prescribed carefully due to their frequent side effects and dependence, orthopedic surgeons are reported to be the fourth-highest prescribers of opioids [7]. It has been reported that femoral neck fracture is an independent predictor of prolonged opioid use in patients undergoing total hip arthroplasty, and it may be associated with increased risk of infection, dislocation, and aseptic revision [8]. Therefore, it is important to recognize other effective pain relief options in patients with hip fractures to decrease the required opioid administration and subsequently decrease its’ adverse events. Based on a systematic review by Abu-Setta et al. (which included published articles in 25 electronic databases in eight languages), there are no evidence-based guidelines available for hip fracture pain management. They also mentioned that only nerve blockade provided moderate evidence in reducing acute pain after hip fracture, and evidence was insufficient for supporting or rejecting the cost-benefit ratio of other pain relief intervention methods (including spinal anesthesia, systemic analgesia, traction, multimodal pain management, and neurostimulation) [9]. Most of the pain management techniques discussed in this systematic review and in the literature are focused on post-operative pain [9, 10].

Wennberg et al. reported that supplementation with low dose fascia iliaca compartment block, in addition to analgesia (intravenous morphine and oral paracetamol) provided better preoperative pain relief in 27% of patients with hip fractures within two hours compared with placebo [11]. Aprato et al. suggested that intraarticular hip injections provided even better preoperative pain management than fascia iliaca compartment block in patients with intracapsular hip fractures [12].

Multiple studies in the literature have reported the neutral effect of skin traction application on preoperative pain relief in patients with hip fractures [1, 4, 13–18]. In a Cochrane review done by Handoll et al. in 2011, it was reported that in the 11 clinical trials available in the literature, there was no evidence regarding the effectiveness of skin-traction in the different types of hip fractures noted. However, this study also mentioned that their data regarding the advantages of traction in specific types of hip fractures and other complications of traction use was inconclusive [1]. In another systematic review by Sammut et al., it was mentioned that among the only five eligible randomized clinical trials published between 2011 and 2020 on this subject, the quality of evidence is weak to make any definite conclusion. Therefore, a proper, detailed randomized controlled trial on the subject was recommended [19].

Despite this evidence, skin traction is routinely applied in many hospitals as a preoperative pain management technique [20, 21]. It accompanies complications including mechanical shearing, allergy to the strapping, and blockage of blood supply to the affected limb in addition to the usual risk of hip fracture related hospitalization (deep vein thrombosis (DVT), pulmonary embolism (PE), urinary tract infection (UTI), delirium). Due to its common use, the medical system’s limited resources, its complications, and the importance of pain relief in this group of patients, the skin traction’s questionable role in pain management must be re-evaluated for this group of patients.

To the best of our knowledge, skin traction’s effect on pain relief in patients with specifically intertrochanteric fractures (as the most common type of hip fractures) has been discussed in only two other studies in the literature [22, 23]. Both studies found effective preoperative pain alleviation in 24 h post-admission measurements; however, no difference in the administered analgesics between the two groups was observed. The results of these two studies is not in concordance with other mentioned studies on pain relief effect of skin tractions on hip fractures (in general), which demonstrates the need for further research on the subject separately.

We analyzed this effect via a parallel randomized controlled trial in this study. The null hypothesis of this research is that there would be no significant differences in terms of the Visual Analogue Scale (VAS) score and administered morphine dosage between the patients who underwent skin tractions and the control group (without skin-traction).

## Methods

This randomized controlled trial is designed, conducted, and written based on the Consolidated Standards of Reporting Trials (CONSORT) 2010 statement [24]. Ethical approval for a single-institution, prospective, parallel randomized controlled trial with an allocation ratio of 1 was obtained from the university’s ethics committee. The project was registered in the Iranian Registry of Clinical Trials (registration reference: IRCT20180729040636N3). Adult patients admitted to our university-affiliated hospital with stable intertrochanteric hip fractures between the first of August 2020 and the 31st of July 2022 (229 patients) were considered for inclusion in the study. Patients were excluded for the following reasons: Refusal to participate in the study (11 patients), accompanying other fractures (including neck and subtrochanteric fractures) (four patients), intertrochanteric fractures with subtrochanteric extensions (two patients), oblique fractures of the proximal femur (two patients), neurologic issues (12 patients), drug addiction (five patients), and scars or swelling or vascular problems at the skin traction application site (one patient) (twenty-six patients in total).

Patients who consented to the study (192 patients) were then randomized to two groups by a medical student (S.KH.) who used random allocation software with an allocation ratio of one [25]. The allocated numbers were written on paper and hidden in envelopes with a numerical sequence written on the envelopes. The envelopes were sealed until the patients’ admission. After the patients’ admission, one orthopedic resident (M.S.), unaware of the allocation sequence, opened the envelope and allocated each patient to a group. Ninety-seven patients in group A underwent skin traction application, and in 95 patients in group B, a pillow was put beneath the knee of the fractured side. Routine pre-operative prophylactic antithrombotic therapy was administered to all the patients.

**Fig. 1:**
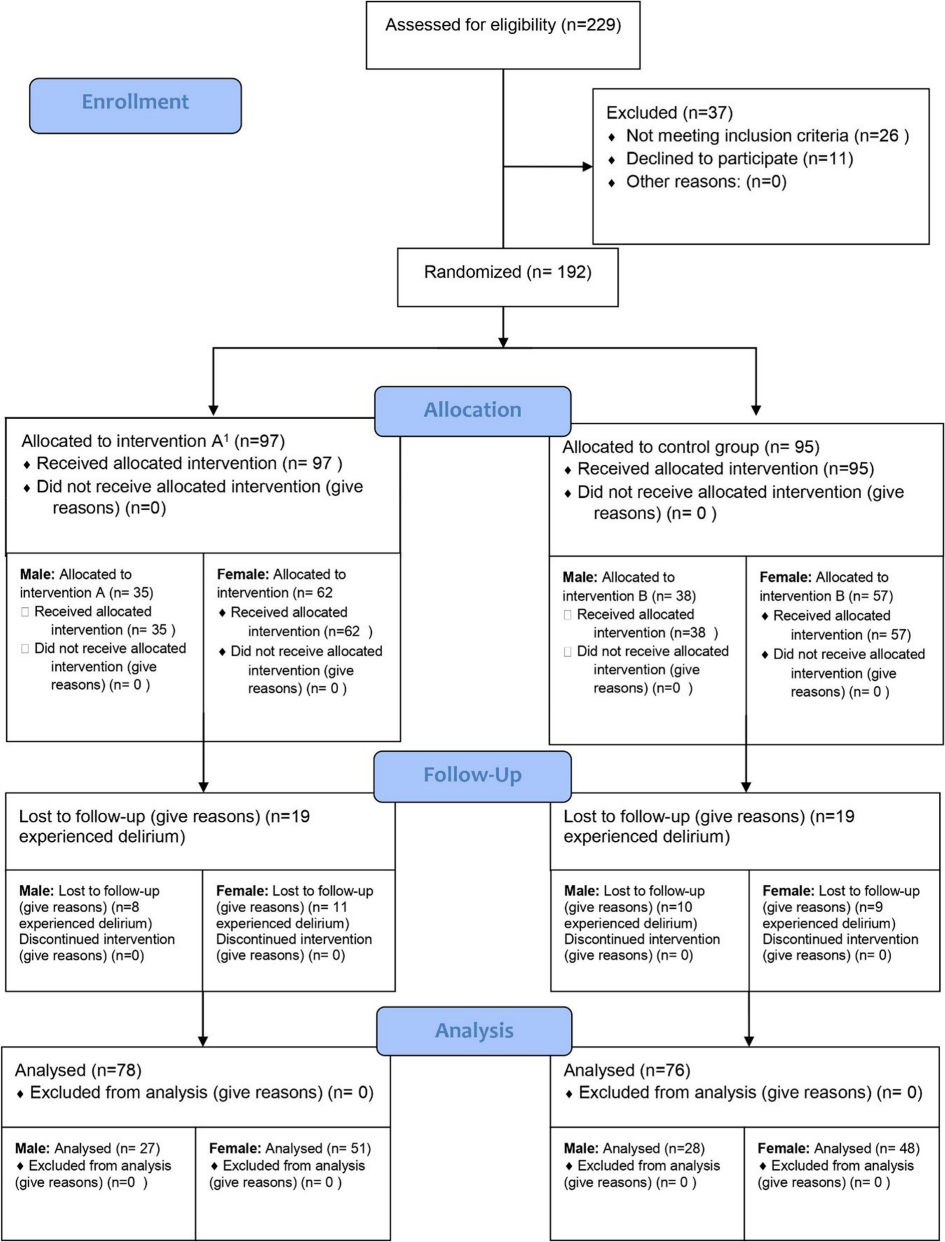
CONSORT flow diagram depicting the number of patients allocated to each group of patients at different study steps

The whole procedure was explained to the patients to obtain written consent. Because skin traction is impossible to hide from both the patients and the medical staff, patients and medical staff were not blinded to the study. However, as traction was removed before the patients’ entrance to the operating room, the surgeon (M.M.) was blinded and was unaware of the group to which the patient was allocated before the surgery. One surgeon (M.M.) for all the patients performed the surgery. Two different surgeries were performed for the patients: extramedullary fixation (dynamic hip screw) and intramedullary nail (Gamma nail) fixation. Also, all the patients were followed up (measuring VAS scores and documenting morphine dosage and any complications) by one orthopedic resident (M.S.) and one trained nurse (T.R.) to reduce the potential performance bias. The data analyst of the study was also blinded by not mentioning which group of the patients underwent skin-traction application. Patients were excluded from the study if they refused to continue the trial at any stage. After undergoing surgery, thirty-eight patients experienced postoperative delirium. These patients were also excluded from the study as their altered level of consciousness could affect their reported VAS scores. In the end, the data of seventy-eight patients in group A and seventy-six patients in group B were entered for the final statistical analysis (Fig. 1).

**Fig. 2:**
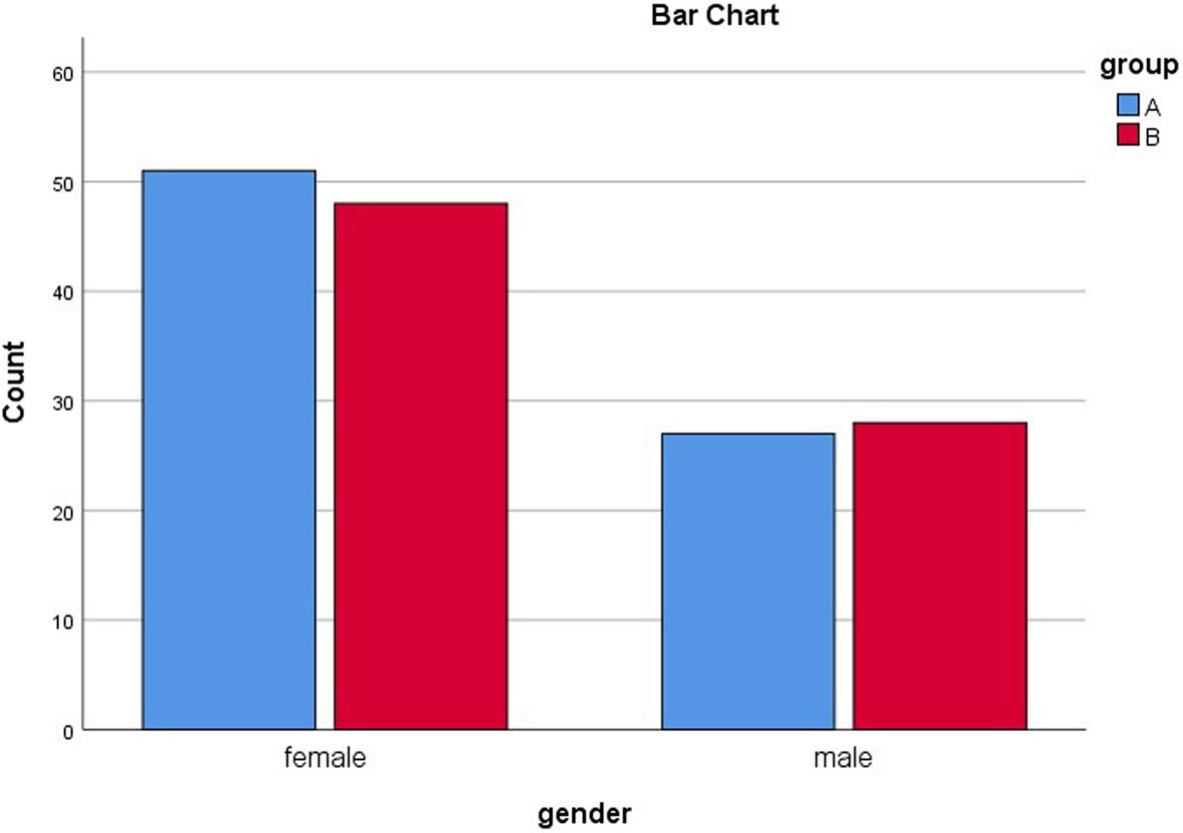
Bar chart comparison between the two groups regarding gender. In group **A** (blue), patients underwent traction application, and in group **B** (red), patients did not undergo traction application

**Fig. 3:**
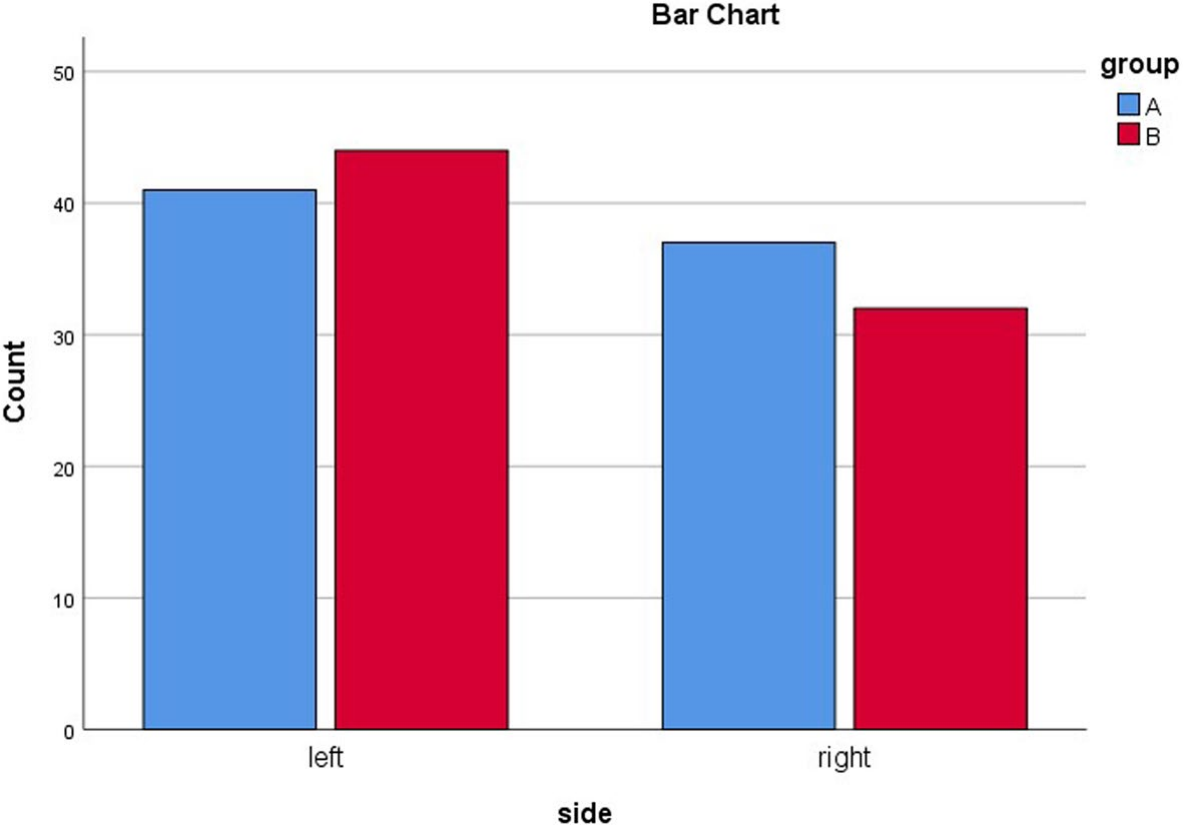
Bar chart comparison between the two groups regarding the fracture side. In group **A** (blue), patients underwent traction application, and in group **B** (red), patients did not undergo traction application

Table 1 demonstrates the statistical comparisons of demographic and non-demographic factors between the two groups. As can be seen in Table 1, the distributions for the mentioned numerical variables were sufficiently normal for conducting a t-test (i.e., skew <|2| and kurtosis <|9.0|) [26]. Based on independent samples t-test results, there were no significant differences between the two groups regarding age (*P* = .75), the mean time from injury to admission (*P* = .08), and the mean time from admission to surgery (*P* = .95). Chi-square test demonstrated that there were no significant differences between the two groups in terms of gender (*P* = .77), and the side of fracture (*P* = .51) (Table 1). Figures 2 and 3 provide bar chart comparisons of age, and fracture side between the two groups.

**Table 1:** The comparison of patients’ characteristics between the two groups

Variable	Total patients (*n* = 154)	With Traction (*n* = 78)	Without traction (*n* = 76)	F/X²	*P*-value
Age (years), (M ± SD) (range)	70 ± 10.2 (40–92)	70 ± 9.8 (40–91)	71 ± 10.6 (46–92)	2.32	0.83
Gender (%)					
Male	55	27	28	0.08	0.77
Female	99	51	48		
Side (%)					
Right	69	37	32	0.44	0.51
Left	85	41	44		
Time from injury to admission (hours) (M ± SD) (range)	6.8 ± 2.3 (1.0-13.3)	7.1 ± 2.4 (1.0-13.3)	6.5 ± 2.2 (1–12)	0.14	0.08
Duration from admission to surgery (days) (M ± SD) (range)	5.6 ± 1.4 (2–9)	5.6 ± 1.3 (3–9)	5.6 ± 1.5 (2–9)	0.63	0.90

The severity of the pain was measured based on VAS scores in patients within the first hour of diagnosis before skin traction application, two hours before the operation (after skin traction application and before patients’ transfer to the operating theater), and 24 h after the procedure. The study’s primary outcome was the effect of skin traction application on the VAS score of patients with intertrochanteric fractures. The secondary outcome of this study was the effect of skin traction on the mean administered intravenous morphine dosage for these patients per day.

The sample size was calculated to be at least 42 in each group using the confidence interval of 95% and the power of study of 90% using the following formula [27]:

$$\mathrm n=\left({\mathrm Z}_{\mathrm\alpha/2}+{\mathrm Z}_{\mathrm\beta}\right)^2\ast\left({\mathrm\sigma}_1^2+{\mathrm\sigma}_2^2\right)/\left(\mu_2\;-\mu_1\right)^2$$

Where α = 0.05, β is the conventional multiplier for power (90% in our study). Z_α/2_ is the constant set by convention according to the accepted α error with a 95% confidence interval (1.96 in our study). Z_β_ is the constant set by convention according to the power of the study. As the power of our study is 90%, Z_β_ would be 1.28.σ_1_ is the standard deviation (SD) in the group without traction (0.9), and σ_2_ is the SD in the group with skin traction (0.8), µ_1_ is the mean of VAS scores in the group without traction (3.3) and µ_2_ is the mean of VAS scores in the group with skin traction (2.7). We used the reported mean VAS scores in Rasi et al’s study for calculating our sample size; the two means were compared using the independent samples t-test [22].

Statistical analysis was done using SPSS version 26, Chicago. Independent samples t-test, chi-square test, and repeated measures Analysis of Variance (ANOVA) tests were used for the statistical analysis. A *P-value* of less than 0.05 was considered significant.

## Results

**Fig. 4:**
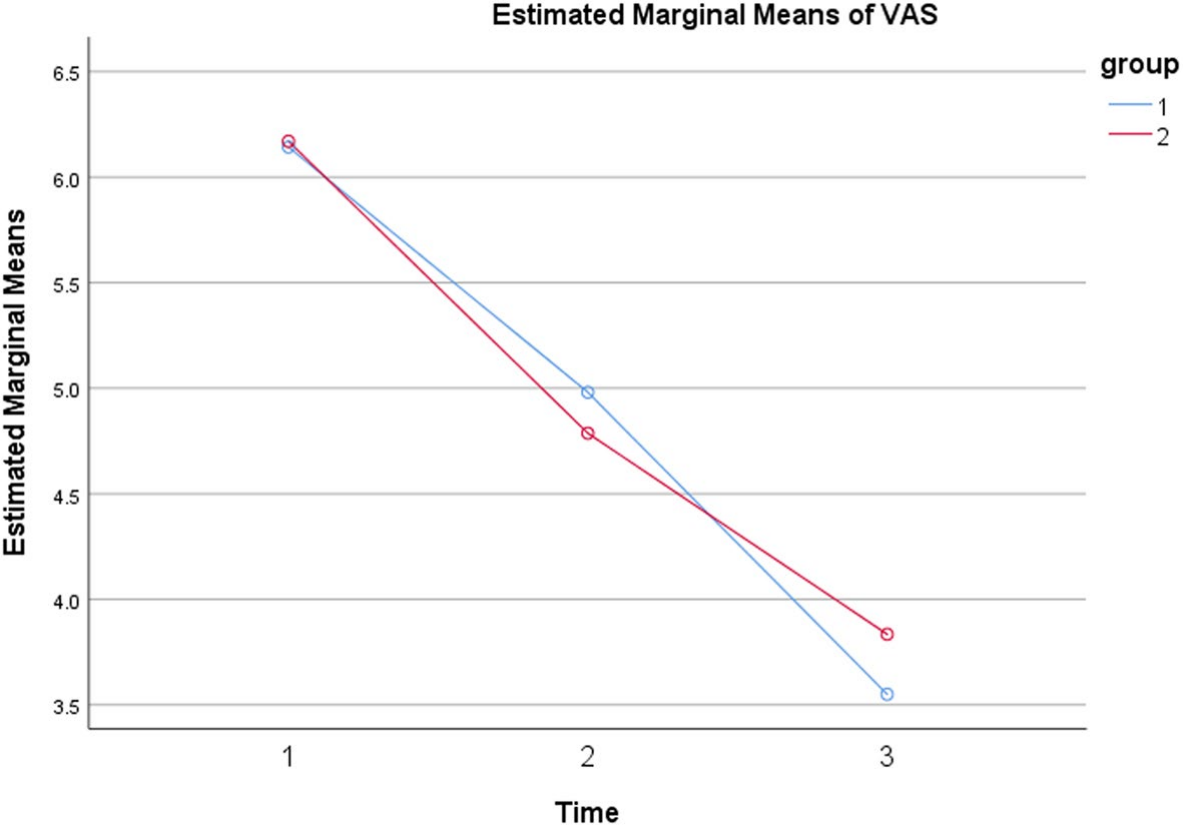
VAS score measurements over time in two groups. As shown, in both groups pain relief was obtained. In group A (blue), patients underwent traction application, and in group B (red), patients did not undergo traction application

The mean VAS scores between the two groups were not significantly different (Table 2). Also, using the repeated measures ANOVA test, the following results were obtained: Mauchly’s test indicated that the sphericity assumption was met (*P* = .21). The multivariate test demonstrated that the main effect of pain was statistically significant, Wilks’ lambda = 0.347, F (2,151) = 142.3, *P* < .001. In other words, the VAS score of the patients decreased significantly in both groups over time (Fig. 4), meaning that, in both groups, patients experienced significant pain relief after the surgery. This effect was NOT qualified by any pain^✕^group interaction, Wilks lambda = 0.978, F (2,151) = 1.691, *P* = .19. Indicating that there was no effect for group on VAS measurements.

**Table 2:** Comparison of VAS measurements between patients with traction and patients without traction

	Total patients (*n* = 154)	Patients with traction (*n* = 78)	Patients without traction (*n* = 76)	*F* value	*P-*value Between the two groups
P1^a^ (Mean ± SD^b^) (range)	6.2 ± 1.4 (2.8–10)	6.1 ± 1.6 (2.8–10)	6.2 ± 1.1 (3.9–9.2)	10.8	0.90
P2^c^ (Mean ± SD) (range)	4.9 ± 1.1 (2.5-7.0)	5 ± 1.0 (2.5-7.0)	4.8 ± 1.0 (2.5-7.0)	0.19	0.24
P3^d^ (Mean ± SD) (range)	3.7 ± 1.3 (0.4–6.6)	3.6 ± 1.2 (0.6–6.6)	3.8 ± 1.3 (0.4–6.5)	0.06	0.16
*P*-value within each group over time	< 0.001	< 0.001	< 0.001		

^a^P1: Mean VAS score in the first hour of admission

^b^SD: Standard Deviation

^c^P2: Mean VAS score 2 h before the surgery

^d^P3: Mean VAS score 24 h after the surgery

The difference in the mean administered morphine dosage in milligrams at different stages of hospitalization per day (*P* = .66) and the duration of surgery (*P* = .95) between the two groups was not significantly different (Table 3).

**Table 3:** Mean morphine dosage in milligrams administered per day and the mean duration of the surgery in two groups and all patients

Variable	Total patients (*n* = 154)	With traction (*n* = 78)	Without traction (*n* = 76)	*F* value	*P*-value
Administered morphine in milligrams per day (M ± SD) (range)	6.5 ± 2.7 (0.8–12.8)	6.6 ± 2.6 (0.8–12.8)	6.4 ± 2.7 (1.5–12)	1.58	0.66
Duration of the surgery(hours) (M ± SD)(range)	1.4 ± 0.5 (0.3–2.9)	1.4 ± 0.5 (0.5–2.9)	1.4 ± 0.5 (0.3–2.8)	0.007	0.95

In terms of complications, the difference between the two groups regarding the number of patients experiencing pressure ulcers (*P* = .54), DVT (*P* = .90), PE (*P* = .84) and UTI (*P* = .66) were not statistically significant (Table 4). In addition, as mentioned in the methods section, 19 patients in the traction group, and 19 patients in the non-traction group experienced postoperative delirium, these patients were excluded from the study as their reported VAS scores could be compromised by their delirious state. No other complications were seen in the two groups of patients.

**Table 4:** Comparison of complications seen between both groups

Variable	Total patients (*n* = 154)	With Traction (*n* = 78)	Without traction (*n* = 76)	X²	*P*-value
Pressure ulcers	23	13	10	0.37	0.54
DVT	31	16	15	0.01	0.90
PE	17	9	8	0.04	0.84
UTI	18	10	8	0.2	0.66

## Discussion

In our study, no significant difference was found in VAS score values and the mean morphine dosage administered per day between the patients with intertrochanteric fractures who underwent skin traction and the control group without traction. Therefore, in terms of pain alleviation, applying skin traction for these patients was only an extra burden on the patients and the caregivers. This is an important finding, as the standard protocol in many countries is to apply skin traction for all patients with intertrochanteric fractures [20, 21].

Before the introduction of fixation devices, treatment for intertrochanteric fractures was completely non-operative, consisting of prolonged bedrest (10 to 12 weeks) in traction until fracture healing occurred. It goes without mentioning that this approach was time-consuming and accompanied different complications, including decubitus ulcers, joint contractures, thromboembolic complications, and increased mortality rate. Since then, and with the introduction of fixation devices, there have been significant improvements in the management of intertrochanteric fractures [28]. However, preoperative traction application (skin or even sometimes skeletal) has still remained in the treatment protocols of these types of fractures in many countries (including ours) over time. In addition, due to the significant reported differences in pain thresholds between different races [29], we hypothesized that the results of this research might be different from other studies from other regions.

Interestingly, no explanation regarding the rationale behind skin traction application for patients with specifically intertrochanteric fractures has been given to the best of our knowledge.

There are, however, several theories in the literature. One theory, in patients with proximal femoral fractures, is that the skin-traction application decreases the chance of instant pain and future non-union and avascular necrosis (AVN) by preventing the external rotation of the fractured site, the occlusion of the vascular supply, and the risk of tamponade. It has also been suggested that patients would have a better range of motion in the no traction group, allowing them to move their legs in a position with the slightest intracapsular pressure and, therefore, less pain [13, 18, 30]. On the other hand, using digital subtraction angiography, Xiao et al. reported that skin traction might impair the blood perfusion to the femoral head and could be one of the major causes of AVN in patients with femoral head fractures [31].

based on a Cochrane review article done by Handoll et al., a well-designed clinical trial is needed, especially for specific fracture types, to evaluate the cost-benefit ratio for traction application in patients [1]. In a review article on lower extremity traction written by Matullo et al., it was concluded that despite its’ steady use throughout medicine, traction application lacks relevant scientific evidence [32].

Intertrochanteric fractures are more common in patients ageing 65 and older. Pharmackokinetics and pharmacodynamics are more complex in elderly patients and they usually experience higher peak effect and longer duration of actions of opioids. This could potentially lead to higher complication rates in terms of delirium, constipation, pulmonary suppression, dependence, etc. [33]. Because of the mentioned adverse events, physicians might be reluctant to prescribe opioids for these patients, and patients’ pain might not be controlled effectively. On the other hand, both opioid narcotics and uncontrolled perioperative pain have been reported to be associated with increased incidence of delirium, and consequently poorer functional outcomes [34–36]. Therefore, the addition of another pain control method would help with less opioid administration and thus decreased opioid related adverse events, and better pain control at the same time.

In addition, most of the studies on pain management in these patients are focused on the post-operative pain, as if patients’ preoperative pain is not recognized as serious pain. While in a recent study conducted by Unneby et al. even though all of the patients received femoral nerve blockades and proper intravenous analgesics, the preoperative pain perceived by patients was describes as “Hovering between heaven and hell” meaning that their pain intensity ranged from having no pain to the worst pain they had ever experienced [37]. They also reported that some patients did not remember their pain or the pain management they had received due to their memory loss; therefore, having no verbal complaint regarding pain does not necessarily imply the absence of pain in geriatric patients and proper pain management options with least possible adverse events must be introduced for these patients.

Rasi et al. conducted a similar clinical trial to ours with a smaller cohort on 40 patients with intertrochanteric fractures. They divided patients into two groups, each consisting of 20 participants with intertrochanteric fractures, and measured their VAS scores multiple times until 24 h postadmission. They reported that only the last VAS score at the end of the 24 h postadmission between the two groups was significant, but interestingly there was no significant difference between the two groups in terms of analgesic administration [22]. However, we believe that our study provides more realistic information regarding the decrease in patients’ pain as we followed up with the patients until one day after surgery; additionally, we studied a higher number of patients showing that our study has a higher power.

Kobayashi et al. performed a retrospective comparative cohort study on 56 patients with intertrochanteric fractures. Eighteen patients underwent skin traction application; for the rest, the skin traction was not applied. In their study, there was no significant difference between the two groups in terms of preoperative pain at 12 h before surgery (measured by Verbal Rating Score (VRS)). Still, the VRS of the patients for whom skin traction was applied were significantly lower than others at 24–60 h after admission. However, interestingly, the two groups did not have any significant differences in terms of analgesic needs based on patients’ requests [38]. We believe that our study provides more reliable results than theirs as our study provides a more reliable methodology (randomized controlled trial). In addition, they used VRS for pain measurement, while most current studies on preoperative pain relief in patients with hip fractures are based on VAS measurements.

Rosen et al. randomly allocated 100 patients (55 patients with femoral neck fractures and 45 with intertrochanteric fractures) into skin-traction and pillow placement groups. Their study noted better immediate pain relief in the group without traction. On the other hand, they found out that if patients had mean initial VAS scores of more than five, the patients who underwent skin traction experienced slightly more pain reduction [39]. However, we believe that our results differed from Rosen et al.’s study as our patients experienced more pain at the time of diagnosis compared to their patients (the mean VAS score of our patients within the first hour of diagnosis was 6.2 ± 1.4, which was higher than all the reported VAS scores in their study). This could be because of the difference in pain conception due to racial differences, emphasizing the importance of conducting this trial in our region again.

Saygi et al. divided 108 patients with hip fractures (intertrochanteric fractures and collum femoris fractures) into three groups: in group one, two-kg skin traction was applied; in group two, skin traction without weight was applied; and in group three, a pillow was put beneath the fractured limb. They evaluated the traction’s placebo effect, and better pain relief was obtained in the group with the placebo. They believed their result was due to semiflexion and external rotation and the placebo effect of the without-weight traction kit [4]. Their findings are valuable as they offer the benefits of the placebo effect of the non-weighing traction on patients’ pain relief. We think skin traction might be helpful in cases where the operation is delayed due to the background disease of the patient, and there is no alternative option for the patient’s condition but to use a placebo.

We also measured the postoperative VAS scores, which were not reported in either of the mentioned studies, as we hypothesized that the placebo effect of skin traction might affect the pain perception in patients postoperatively. Interestingly, the postoperative VAS scores in both groups were significantly lower than the preoperative measurements. This can be because of the fixation of the fracture, postoperative analgesics administered to the patients or the mental relief for them undergoing the ultimate treatment. It has been suggested that patients’ satisfaction does not always reflect the quality of pain management but rather patients’ expectations and perceptions [10]. It has also been reported that a positive relationship exists between patients’ satisfaction and the severity of side effects while managing postoperative pain with analgesics [40]. Therefore, our study’s reported postoperative VAS scores might reflect on the patients’ satisfaction with undergoing surgery (as their definite treatment) rather than their pain.

Anderson et al. studied 252 patients with different types of proximal femur fractures. They randomly allocated the patients to skin traction and the control group with a nurse-free bed (method of nursing the injured limb was not specified in this study). They reported no differences in pain suffered, analgesia injection, frequency of pressure ulcers, or ease of operations between these two groups [13]. We found their methodology inspiring and evaluated if the results would be the same in patients with only intertrochanteric fractures.

Over the years, multiple studies reported that skin traction might not affect the severity of the pain and the dosage of required analgesics for patients with different types of hip fractures [13–16, 18, 41, 42]. However, our study is among the few controlled trials in the literature that has specifically evaluated the effect of skin traction on the prescribed morphine in patients with intertrochanteric fractures. The mean morphine dosage administered per day in our study was not significantly different between the two groups. This was in concordance with the results of the VAS score analysis. Therefore, no adjustment of the two effects was needed. Resell et al. studied 153 patients with displaced cervical and trochanteric hip fractures with the same study method as ours and found out that the patients in the traction group even needed more analgesic dosage, although not significantly different [17]. This study was repeated on 123 patients comparing the skin-traction effect with the Lasse pillow effect on pain relief. There was no clinically significant difference in pain relief or analgesic usage between the two groups [16]. Although both methodologies were thorough, in our humble opinion, their results could not be generalized to both fracture types they included in their study. As mentioned, we exclusively studied patients with intertrochanteric hip fractures.

There is an agreement in the literature that skin traction does not cause short-term severe complications for patients with different types of hip fractures. In addition to the surgery and hospitalization related complications (UTI, DVT, PE), pressure ulcers, mechanical shearing, allergy to the strapping, and blockage of blood supply to the affected limb are reported as direct complications of skin traction [1, 4, 15, 18]. In our study, no significant differences in complication rates between the two groups were noticed.

In addition, the mean time from admission to surgery in all the patients (which in our study equals to the length of time that patients were in skin traction) was 5.6 ± 1.4 days, and there was no significant difference between the two groups in terms of length of time from admission to surgery.

Early surgery has been reported to be associated with fewer in-hospital complications, improved functional outcomes, increased return to independent living, and decreased overall mortality [43, 44]; however, it is important to find the correct timing for the operation, and make a distinction between complications which could happen due to the delay in surgery, and the complications that are potential causes for the delay to surgery.

Beaupre at al. reported that 20 to 40 h of delay in surgery was associated with highest survival rate in patients older than 80 years of age; however, they mentioned that in patients who are 60 years old or younger, surgery can be delayed without any decrease in mortality rate. Therefore, they suggested that sub-prioritization of patients should be based on patients characteristics, rather than the national benchmarks [45]; Even though their study results are valid, we assume that mortality rate should not be the only predictive factor for determining the proper time to surgery, and other potential minor and major complications should also be considered as well. Lefaivre et al. reported that in their study, delay to surgery of more than 24 h was accompanied with minor complications, while more than 48 h of delay to surgery significantly increased the risk of major medical complications [46]. On the other hand, Grimes at al. performed a retrospective cohort study on 8383 patients with deferent types of hip fractures and reported that longer time to surgery was only associated with increased risk of decubitus ulcer formation and no short-term or long-term (up to 18 years) mortality after adjusting for other medical problems; however, in their study, the median time to surgery was 23 h and 92% of patients underwent surgery within 72 h postadmission [47].

In an observational study on 83,727 patients with hip fracture performed by Leer-Salvesen et al., it was reported that delay to surgery of more than 48 h was associated with increased three day mortality, and 1 year mortality. Also, patients with delay to surgery of more than 24 h, experienced more intraoperative complications. In their study, the mean pre-hospital delay was six hours, and the mean in-hospital delay was 22 h. They reported that there was no significant effect of total delay to surgery as long as the patients underwent medical intervention within 48 h postadmission; however, mortality increased in patients who were operated on after 48 h of admission. Even though they adjusted their results based on patients’ fracture types, they ultimately suggested that preoperative patient stabilization should not be used as an argument to delay surgical intervention [48]. On the other hand, Rijckevorsel et al. reported that longer than 48 h of delay in surgery due to only non-medical reasons, was associated with higher pressure ulcer and UTI; however, delay in surgery was not related to 30-day mortality rate and post-operative length of hospital stay. In their study, patients with surgery delays due to optimizing patients’ medical condition were excluded, as authors claimed that there are previous studies, suggesting that postponement of surgery in such conditions would improve the outcome and would not affect the mortality rate [49, 50].

To improve the surgical outcomes, many countries have established time to surgery benchmarks for patients with hip fractures, which reflect the quality of care. For example, within 48 h and 36 h of admission have been set as the national benchmarks in Canada and the UK respectively. Unfortunately, there is no such national time to surgery benchmarks in our country, which could be one of the reasons behind the observed long delay in surgery in our study. Especially, surgical interventions for patients with stable intertrochanteric hip fractures are not considered urgent, and patients might not be prioritized for immediate surgeries; considering that skin traction and morphine administration are currently the only methods of pain alleviation in our country, prolonging the time to surgery period could potentially cause further delirium and other opioid related complications in these patients. We believe that introducing time to surgery benchmarks in the national hip fracture approach protocols would be a critical step toward improving the surgery outcomes and patients’ satisfaction.

In addition, even though patients with intertrochanteric hip fractures are usually older and more susceptible to experiencing severe forms of COVID-19 due to their background diseases, no prioritization was made for this group of patients in our hospital during the COVID-19 pandemic. Malik-Tabassum et al. performed an observational study on patients with different types of hip fractures in the UK, and compared the patients outcomes during 50 days of COVID-19 pandemic (period C) to two same dates within the past 2 years of the study before the pandemic (periods A and B). They reported that the mean length of inpatient stay during period C was significantly shorter than periods A and B; however, there was no significant difference in complication rates, return to the OR, and 30 day mortality rate, and the proportion of the patients discharged to their pre-admission residence during all three periods. They also mentioned that during period C, a multidisciplinary decision was made to prioritize surgery for patients with hip fractures despite the lack of available operating room’s capacity. In addition, they reported that no member of the on-call structure, junior ward cover, or provision of orthogeriatrc care was redeployed to other specialties [51]; however, in our medical center, almost 20% of the orthopedic residents were redeployed to the COVID-19 centers. Also, in our hospital all of the patients have to be consulted by a cardiologist and an anesthesiologist before surgery, and during the study period, the number of available cardiologists and anesthesiologists were limited as many of them were redeployed to COVID-19 centers. In addition, all the patients had to wait for the COVID-19 PCR test result before undergoing surgery which caused further delay in surgery. We believe that with more proper resource and staff management, time to surgery for this group of frail patients could have been shorter and further studies and newer protocols (for both critical and uncritical times) are needed to reduce the time to surgery, and improve the surgery outcomes.

Based on a systematic review written by Abou-Setta et al. most of the common pain control strategies for patients with hip fracture have low to moderate effects, and the evidence on pain management after hip fractures is surprisingly sparse [9]. In this systematic review it was also reported that using nerve blockades (including 3-in-1, combined lumbosacral plexus, fascia iliaca compartment, femoral nerve, lumbar plexus plus sciatic nerve, posterior lumbar plexus, psoas compartment, obturator nerve, epidural and combined blockades) were significantly related to less supplemental pain medication administration and delirium incidence. In addition, nerve blockade and neuraxial anesthesia had similar results in terms of acute pain, use of additional pain medications and delirium. Also, they outlined that adding other agents to plain local anesthesia did not affect the outcomes outside the operating room and increasing dose of local anesthetic for the hip fracture surgery was associated with increased risk of hypotension [9]. Nerve blockades are available in most medical centers; however, they are not used clinically because of a common belief that the additional required time and effort does not outweigh their benefits. However, considering the lack of enough evidence supporting the effect of skin tractions on pain relief in patients with intertrochanteric fractures, we suggest that the focus of the medical approach to pain management shifts toward alternative and more effective pain relief options including nerve blockades.

Our study had some limitations. Due to the COVID-19 pandemic, we decreased the number of VAS measurements to protect our personnel and patients from unnecessary contacts. In addition, even though everyday emphasis was made on not using any other analgesics, there was no controlling for the use of non-opioid/narcotic pain relief medications during both pre- and post-operative periods by patients separately. Also, although we evaluated many patients with specifically intertrochanteric fractures, we did not separate patients with different types of surgery approaches for intertrochanteric fractures (extramedullary fixation (dynamic hip screw) and intramedullary nail (Gamma nail) fixation surgeries); however, we compared the duration of surgery for both groups. As mentioned in the results section, their difference was not statistically significant, meaning the two groups probably had the same heterogeneity in terms of intertrochanteric fracture surgery types. In addition, we did not have enough time to follow up with the patients to report the surgery’s long-term complications. This, however, seems to be a limitation in many previous studies [1, 4, 13, 16, 18, 22, 38, 39]. We recommend that future studies focus on using better preoperative pain management techniques (including nerve blockades) in patients with intertrochanteric fractures with longer follow-ups.

## Conclusion

In conclusion, in our research, skin traction application affected neither the patients’ mean VAS scores nor the mean morphine dosage administered daily in patients with isolated intertrochanteric fractures. Considering the limited hospital time and resources and its burden on both patients and medical staff, we recommend removing skin traction from the routine protocols for patients with intertrochanteric hip fractures, and considering other potential options for patients’ pain relief instead.

## Data Availability

The datasets generated and/or analyzed during the current study are not publicly available due to patients’ privacy but are available from the corresponding author on reasonable request.

## Abbreviations

AVN: Avascular Necrosis
DVT: Deep Vein Thrombosis
PE: Pulmonary Embolism
UTI: Urinary Tract Infection
VAS: Visual Analogue Scale
OR: Operating Room
